# Hyperoxia Provokes Time- and Dose-Dependent Gut Injury and Endotoxemia and Alters Gut Microbiome and Transcriptome in Mice

**DOI:** 10.3389/fmed.2021.732039

**Published:** 2021-11-17

**Authors:** Yunhang Li, Yuanfa Tao, Jingyu Xu, Yihuai He, Wen Zhang, Zhigang Jiang, Ying He, Houmei Liu, Miao Chen, Wei Zhang, Zhouxiong Xing

**Affiliations:** ^1^Department of Cardiology, Affiliated Hospital of Zunyi Medical University, Zunyi, China; ^2^Department of General Surgery, Renmin Hospital of Wuhan University, Wuhan, China; ^3^Department of Gastroenterology, Affiliated Hospital of Zunyi Medical University, Zunyi, China; ^4^Department of Infectious Diseases, Affiliated Hospital of Zunyi Medical University, Zunyi, China; ^5^Department of Critical Care Medicine, Affiliated Hospital of Zunyi Medical University, Zunyi, China; ^6^Department of Statistics, Zunyi Medical University, Zunyi, China; ^7^Department of Endodontics, Affiliated Stomatological Hospital of Zunyi Medical University, Zunyi, China

**Keywords:** oxygen therapy, hyperoxia, gut injury, gut microbiome, *Enterobacteriaceae*, toll-like receptor-4, NOD, innate immunity

## Abstract

**Background:** Oxygen therapy usually exposes patients to hyperoxia, which induces injuries in the lung, the heart, and the brain. The gut and its microbiome play key roles in critical illnesses, but the impact of hyperoxia on the gut and its microbiome remains not very clear. We clarified the time- and dose-dependent effects of hyperoxia on the gut and investigated oxygen-induced gut dysbiosis and explored the underlying mechanism of gut injury by transcriptome analysis.

**Methods:** The C57BL/6 mice were randomly divided into the control group and nine different oxygen groups exposed to hyperoxia with an inspired O_2_ fraction (FiO_2_) of 40, 60, and 80% for 24, 72, and 168 h (7 days), respectively. Intestinal histopathological and biochemical analyses were performed to explore the oxygen-induced gut injury and inflammatory response. Another experiment was performed to explore the impact of hyperoxia on the gut microbiome by exposing the mice to hyperoxia (FiO_2_ 80%) for 7 days, with the 16S rRNA sequencing method. We prolonged the exposure (up to 14 days) of the mice to hyperoxia (FiO_2_ 80%), and gut transcriptome analysis and western blotting were carried out to obtain differentially expressed genes (DEGs) and signaling pathways related to innate immunity and cell death.

**Results:** Inhaled oxygen induced time- and dose-dependent gut histopathological impairment characterized by mucosal atrophy (e.g., villus shortening: 80% of FiO_2_ for 24 h: *P* = 0.008) and enterocyte death (e.g., apoptosis: 40% of FiO_2_ for 7 days: *P* = 0.01). Administered time- and dose-dependent oxygen led to intestinal barrier dysfunction (e.g., endotoxemia: 80% of FiO_2_ for 72 h: *P* = 0.002) and potentiated gut inflammation by increasing proinflammatory cytokines [e.g., tumor necrosis factor alpha (TNF-α): 40% of FiO_2_ for 24 h: *P* = 0.003)] and reducing anti-inflammatory cytokines [Interleukin 10 (IL-10): 80% of FiO_2_ for 72 h: *P* < 0.0001]. Hyperoxia induced gut dysbiosis with an expansion of oxygen-tolerant bacteria (e.g., *Enterobacteriaceae*). Gut transcriptome analysis identified 1,747 DEGs and 171 signaling pathways and immunoblotting verified TLR-4, NOD-like receptor, and apoptosis signaling pathways were activated in oxygen-induced gut injury.

**Conclusions:** Acute hyperoxia rapidly provokes gut injury in a time- and dose-dependent manner and induces gut dysbiosis, and an innate immune response is involved in an oxygen-induced gut injury.

## Introduction

Supplemental oxygen is the cornerstone of intensive care medicine, and it usually exposes patients to excess oxygen, or hyperoxia (1). Hyperoxia induces oxidative stress and leads to multiple organ injuries and high mortality in patients who are critically ill (2–4). Gut homeostasis plays a pivotal role in critical illness, and gut injury associated with gut barrier failure is common in intensive care units (ICUs) (5). Gut barrier dysfunction leads to the absorption of lipopolysaccharide (LPS) from bacteria in the gut lumen into the bloodstream, resulting in endotoxemia. Gut leakage plus endotoxemia are the origins of sepsis and multiple organ dysfunction syndromes (MODS) (6). A few studies have indicated that the gut is also vulnerable to hyperoxia (7–9), but the impact of acute hyperoxia on the gut is not very clear. There are few data evaluating the impairment induced by hyperoxia under different concentrations of oxygen and durations of exposure. In this study, we explored the time- and dose-dependent gut injury induced by acute hyperoxia in a mouse model.

The gut is home to trillions of complex microorganisms. Gut microbiota plays a fundamental role in human health and diseases and in the development of critical illnesses (10, 11). Oxygen shapes the physiology and structure of gastrointestinal microbes (12, 13). Recent studies indicate that hyperoxia alters the composition of the gut microbiome (14–17). However, these findings are limited as a result of great heterogeneity. We attempted to explore the impact of hyperoxia on the gut microbiome in a mouse model with a relatively large sample size, elucidating the qualitative and quantitative effects of hyperoxia.

Gut microbes contribute to the development and integrity of the gut mucosal system through innate immunity, which involves the recognition of pathogen-associated molecular patterns (PAMPs) by pathogen-recognition receptors (PPRs) (18, 19). Toll-like receptors (TLRs) and nucleotide-binding oligomerization domain (NOD)-like receptors are the most important PPRs that are activated by LPS or peptidoglycan (PGN) from bacteria (20, 21). The gut microbiota modulates the phenotype of gut injury induced by environmental factors through TLR and NOD-like receptor signaling pathways (22, 23). Last, we performed gut transcriptome analysis in mice exposed to hyperoxia to explore the underlying mechanism of oxygen-induced gut injury, focusing on the innate immune response.

## Materials and Methods

The research was carried out following the ARRIVE guidelines (www.arriveguidelines.org).

### Mice and Hyperoxia Exposure

The mouse experiments were performed in compliance with the National Institutes of Health guidelines and were approved by the Institutional Animal Care and Use Committee at Zunyi Medical University. Eight-week-old specific-pathogen-free (SPF) C57BL/6J male mice (the same strain) were purchased from GemPharmatech Company (Nanjing, China) and were housed under barrier-maintained SPF conditions. All mice had free access to the same water and chow (Xietong Company, Nanjing, China) and were reared in temperature- and humidity-controlled colony rooms, with 12 h shift of the light-dark cycle. All devices for hyperoxia modeling were preconditioned by radiation disinfection before entering the colony units. In each experiment of the study, the control and oxygen groups were kept under the same housing conditions in the same colony room.

#### Experiment 1

A total of 60 mice were recruited and were randomly assigned to the control group (*n* = 6) and nine different oxygen groups (*n* = 6) on the body-weight stratification. The control group was reared under room air (21% of FiO_2_) for 0 h as the baseline. The nine oxygen groups were exposed to medical oxygen (Xuqin, Wuhan, China) with an inspired O_2_ fraction (FiO_2_) of 40, 60, and 80% for 24, 72, and 168 h (7 days), respectively. The control and the nine oxygen groups were reared in 10 independent cages (*n* = 6), respectively. The 10 cages were simultaneously put into three hyperoxia chambers (Puhe Company, Wuxi, China) for hyperoxia modeling and one hyperoxia chamber for normoxia modeling. For hyperoxia modeling, the three hyperoxia chambers, each containing three independent cages (duration of exposure: 24, 72 h, and 7 days), were exposed to FiO_2_ of 40, 60, and 80%, respectively. For normoxia modeling, the hyperoxia chamber contains one cage and was exposed to room air (FiO_2_ of 21%) for 0 h.

#### Experiment 2

A total of 34 mice were pooled and randomly divided into the control group (*n* = 18) and the oxygen group (*n* = 16) on the body-weight stratification. The mice in the control group were reared in four independent cages (*n* = 5 in two cages and *n* = 4 in another two cages), and the mice in the oxygen group were reared in another four independent cages (*n* = 4). The control group (four cages) and the oxygen group (four cages) were put into two hyperoxia chambers for normoxia modeling and hyperoxia modeling, respectively. The control group was reared under room air (FiO_2_ of 80%) for 7 days, and the oxygen group was exposed to hyperoxia (FiO_2_ of 80%) for 7 days. The fecal pellets of the animals were collected in biological safety cabinets after exposure to normoxia and hyperoxia for 0, 72, and 168 h (7 d), respectively. The fecal pellets were rapidly frozen into liquid nitrogen for microbe analysis.

#### Experiment 3

To increase the expression of differentially expressed genes (DEGs), we prolonged the exposure of the mice to hyperoxia (FiO_2_ 80%) up to 14 days. A total of 12 mice were randomly divided into the control and oxygen groups (*n* = 6). The oxygen group (one cage) was exposed to hyperoxia for 14 days, and the control group (one cage) was reared under room air for 14 days. Transcriptome analysis was performed to identify marker DEGs and signaling pathways related to oxygen-induced gut injury. Subsequent Western blotting was carried out to verify the levels of essential proteins involved in the signaling pathways of innate immunity (TLR-4 and NOD-like receptor) and cell death (apoptosis).

No mice exposed to hyperoxia died during the experiments. Animals were deeply anesthetized and euthanized. Blood was obtained by retrobulbar venous plexus puncture for the analysis of serum levels of LPS and D-lactic acid. The abdomen was opened by a midline incision, and the distal small intestine (ileum) was harvested for histopathological examinations, biochemical analysis, and transcriptome analysis.

### Histopathological Examinations

The last 2 cm length of the distal intestine was carefully excised and removed. The intestine was fixed overnight in paraformaldehyde, embedded in paraffin, sliced into 5 μm-thick sections, and stained with hematoxylin and eosin. Histopathological examinations were performed by two pathologists blinded to the oxygen and control groups. The sections were carefully viewed with a light Nikon Eclipse CI microscope (Tokyo, Japan) to evaluate the gut morphology. We randomly selected 10 villi and crypts in every mouse and measured the villus height and crypt depth from the baseline to the villus tip and from the baseline to the submucosa, respectively. We also graded the gut injury according to an intestinal mucosal injury scoring system developed by Chiu (24). The images were processed with Image Pro Plus 6.0 (Media Cybernetics, Silver Spring, USA).

### TdT-Mediated DUTP-Biotin Nick End Labeling Assay

A TdT-mediated dUTP-biotin nick end labeling (TUNEL) kit (Roche, Basel, Switzerland) was used for *in situ* detection of apoptosis in the gut mucosa, in accordance with the instructions of the manufacturer. The sections with immunofluorescent staining were examined with a Nikon Eclipse Ti-SR microscope (Tokyo, Japan). We randomly collected six fields per slide to count the apoptotic cells for quantification.

### Western Blotting

The gut tissue was harvested and the proteins were extracted. Western blotting was performed with the primary antibodies for caspase-1 (Boster, Wuhan, China), gasdermin-D (GSDMD) and zonula occludens-1 (ZO-1; Proteintech, Chicago, USA), occludin (Abcam, Cambridge, UK), Fas and Bcl-2 (CST, Shanghai, China), TLR-4 and Myd88 (Affinity, Ancaster, Canada), NOD1 and NOD2 (ABclonal, Wuhan, China), NOD-like receptor family pyrin domain containing 3 (NLRP3) and glyceraldehyde-3-phosphate dehydrogenase (GAPDH; Abcam) as the internal control. The proteins were separated by sodium dodecyl sulfate polyacrylamide gel (8–10%) and were transferred onto polyvinylidene fluoride membranes (Millipore, Bedford, USA). The membranes were incubated with primary antibodies and subsequently incubated with horseradish-peroxidase-conjugated goat anti-rabbit secondary antibodies (KPL, Milford, CT, USA).

### Immunohistochemistry

Immunohistochemical staining was performed by the immunoperoxidase visualization method on the paraffin-embedded intestinal sections. Anti-ZO-1 proteins (Proteintech, IL, USA) and horseradish-peroxidase-conjugated goat anti-rabbit secondary antibodies (SeraCare, Milford, USA) were used as the primary and secondary antibodies, respectively. The immunostained sections were observed with a light Nikon Eclipse CI microscope (Tokyo, Japan) at a magnification of 200×. The mean optical density values of the ZO-1-positive staining of each section were measured with the Image Pro Plus 6.0 (Media Cybernetics, Silver Spring, MD, USA).

### Enzyme-Linked Immunosorbent Assay

The IgM mouse coated ELISA kits (Meimian, Yancheng, China) were used, following the instructions of the manufacturer. The concentrations of serum LPS were quantified to assess endotoxemia. Gut inflammation was explored by measuring intestinal inflammatory cytokines, including lysozyme, D-lactic acid (D-LA), tumor necrosis factor (TNF)-α, interleukin (IL)-1β, IL-6, IL-10, interferon (IFN)-γ, chemokine (C-X-C motif) ligand (CXCL)-1 and 8-Hydroxy-2′-deoxyguanosine (8- OHdG). We determined the absorbance of each solution at 450 nm wavelength and calculated the concentrations from a linear standard curve.

### Microbe Analysis

The bacterial DNA was extracted from the fecal pellets with a Guide S96 DNA kit (Tiangen, Beijing, China), according to the instructions of the manufacturer. PCR amplification was carried out using 16S rRNA primers targeting the variable region V3-V4: 5′-ACTCCTACGGGAGGCAGCA-3′ and 5′-GGACTACHVGGGTWTCTAAT-3′ (25). Sequencing libraries were constructed and paired-ended sequencing was conducted on an Illumina NovaSeq 6000 platform (Illumina, San Diego, CA, USA) at Biomarker Technologies Company (Beijing, China). FLASH (26) (fast length adjustment of short reads to improve genome assemblies), Trimmomatic (27), and UCHIME (28) were used to merge, quality filter, and remove the duplicates of the raw sequences, respectively.

USEARCH was used to cluster the tags into operational taxonomic units (OTUs) with 97% similarity (29), and the identified taxonomy was performed on the basis of the Silva database by the QIIME (30). We used principal coordinates analysis (PCoA) and Unweighted UniFrac distances to evaluate the difference between the control and oxygen groups (e.g., beta diversity). Permutational multivariate analysis of variance (PERMANOVA) in R version 4.0.5 (R Foundation, Vienna, Austria) was used to test significant differences in beta diversity analysis. The linear discriminant analysis (LDA) (31) with effect size measurements was carried out to identify the biomarkers within different groups with a Log10 LDA score of 4 or more. The raw sequencing data of this study were submitted to the Genome Sequence Archive in National Genomics Data Center, China National Center for Bioinformation (32), under project PRJCA006101; accession number CRA004740 that are publicly accessible at https://bigd.big.ac.cn/gsa.

### Transcriptome Analysis

The gut tissue was swiftly removed from the mice and frozen in liquid nitrogen until needed. RNA concentration, purity, and integrity were assessed using NanoDrop 2000 (Thermo Fisher Scientific, Wilmington, NC, USA) and the Agilent RNA Nano 6000 Kit (Palo Alto, USA). Sequencing libraries were generated using NEBNext UltraTM RNA Library Prep Kit (NEB, Ipswich, MA, USA) for Illumina and index codes were added to attribute sequences to each sample. The clustering of the index-coded samples was performed using TruSeq PE Cluster Kit v4-cBot-HS (Illumina, CA, USA). The library preparations were sequenced on an Illumina NovaSeq 6000 platform and paired-end reads were generated and further processed. Analysis of DEGs of the control and oxygen groups was performed using the DESeq2 (33). Genes with adjusted *P* < 0.05 found by DESeq2 were assigned as DEGs. Gene function was annotated based on the Kyoto Encyclopedia of Genes and Genomes (KEGG) database (34), and KEGG pathway enrichment analysis was performed with BMKCloud (www.biocloud.net) online platform. The raw sequence data have been deposited in the Genome Sequence Archive in National Genomics Data Center, China National Center for Bioinformation (32), under project PRJCA005085; accession number CRA004276 that are publicly accessible at https://bigd.big.ac.cn/gsa.

### Statistical Analysis

Statistical analysis was performed using Prism 8.0 (GraphPad, San Diego, USA) and R version 4.0.5 (R Foundation, Vienna, Austria). The data (when normally distributed) of the time- and dose-dependent gut injury were analyzed with a two-way ANOVA followed by Dunnett's *post-hoc* test. One-way ANOVA followed by Dunnett's *post-hoc* test was performed to identify the difference between multiple comparisons vs. control within group effects. Kruskal–Wallis H test followed by the Nemenyi test was used for the analysis of the time- and dose-dependent gut injury scores due to their non-normal distribution. Student's *t*-test (when normally distributed) or Mann–Whitney *U*-tests (when non-normally distributed) was used for the comparison of the relative abundance of bacteria, DEGs, and protein levels between the two groups. All *P* < 0.05 were considered statistically significant.

## Results

### Hyperoxia Induces Time- and Dose-Dependent Gut Histopathological Injury

**Figure 1:**
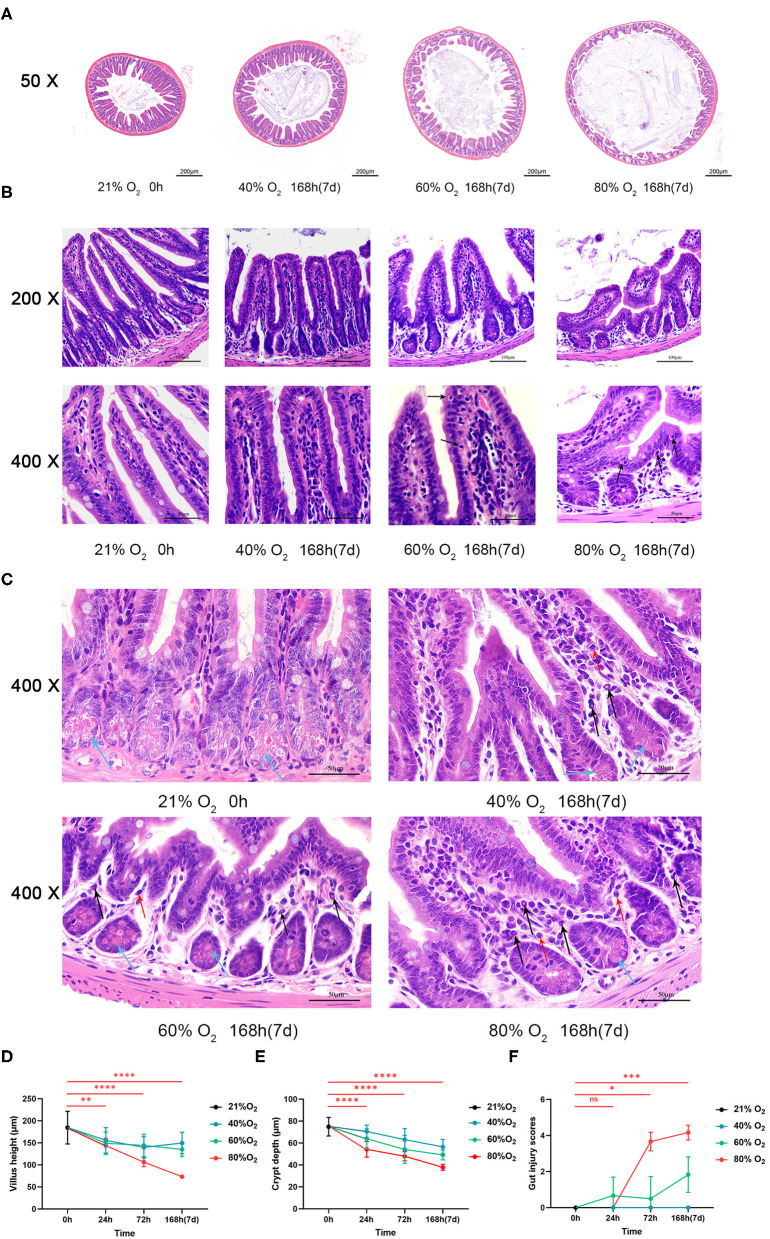
Hyperoxia induces time- and dose-dependent intestinal histopathological injury (*n* = 6). **(A)** Representative intestinal sections from the control group and the oxygen groups (FiO_2_ of 40, 60, and 80%) were exposed for 168 h (7 d) at low magnification (50×). **(B)** Representative intestinal sections from the control group and the oxygen groups (FiO_2_ of 40, 60, and 80%) were exposed for 168 h (7 days) at high magnification (200× and 400×). The mice reared in room air had a normal gut mucosal framework with intact and well-defined villi. There was remarkable intestinal mucosal atrophy in the oxygen groups (FiO_2_ of 60 and 80%) which was featured by villus shortening and intestinal epithelial cell (IEC) degeneration with nucleus shrinkage and pink cytoplasm (black arrows). **(C)** Representative intestinal sections showing inflammatory cell infiltration from the control group and the oxygen groups (FiO_2_ of 40, 60, and 80%) were exposed for 168 h (7 days) at high magnification (400×). There are more infiltration of polymorphonuclear leucocytes (neutrophils, black arrows) and macrophages (red arrows) in the IEC in the oxygen groups vs. the control group. There are less paneth cells (blue arrows) filled with eosinophilic particles at the base of small intestinal crypts in the oxygen groups vs. the control group. **(D)** The graph of the villus height in the control and the oxygen groups. **(E)** The graph of the crept depth in the control and the oxygen groups. **(F)** The graph of the intestinal injury scores in the control and the oxygen groups. *P < 0.05; **P < 0.01; ***P < 0.001; ****P < 0.0001 (*P*-values: 80% of oxygen group vs. the control group).

Representative intestinal sections from the control and oxygen groups exposed for 168 h (7 days) are shown at low magnification (Figure 1A) and high magnification (Figure 1B). The mice reared in room air had a normal gut mucosal framework with intact and well-defined villi. However, hyperoxia (FiO_2_ of 60 and 80% for 168 h) led to the destruction of the intestinal mucosa, showing villus shortening, atrophy, and lodging, and there was a degeneration of the intestinal epithelial cell (IEC) with nuclear shrinkage and pink cytoplasm, indicating apoptosis (Figure 1B, black arrows). In addition, there are more infiltration of polymorphonuclear leucocytes (neutrophils, black arrows) and macrophages (red arrows) and there are less paneth cells (blue arrows) in the intestinal epithelium in the oxygen groups vs. the control group (Figure 1C). In addition, hyperoxia (FiO_2_ of 80% for 7 days) increases the level of lysozyme (*P* < 0.0001; Supplementary Figure 1).

We compared the villus height, crypt depth, and intestinal injury scores in the control group vs. the oxygen groups (*n* = 6 per exposure per time point, Figures 1D–F) and presented the ANOVA and non-parametric analysis in Supplementary Table 1. The villus height was lower after exposure to 80% of oxygen for 24, 72, and 168 h, respectively (*P* = 0.008, *P* < 0.0001, and *P* < 0.0001; Figure 1D). The crypt depth was lower after exposure to 80% of oxygen for 24, 72, and 168 h, respectively (all *P* < 0.0001; Figure 1E). The intestinal injury scores were increased after exposure to 80% of oxygen for 72 and 168 h, respectively (*P* = 0.011 and *P* = 0.0005; Figure 1F). In general, there was gut histopathological injury after exposure to 60% oxygen for 72 and 168 h, respectively.

### Hyperoxia Induces Time- and Dose-Dependent Gut Cell Death

**Figure 2:**
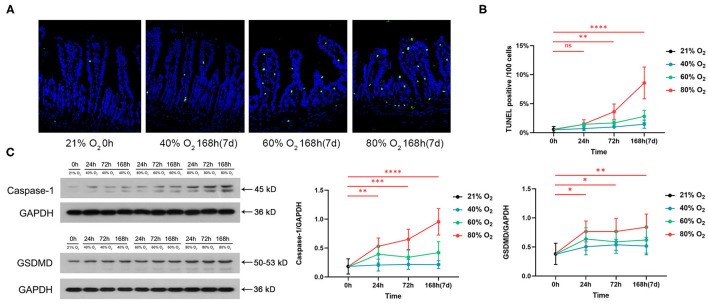
Hyperoxia induces time- and dose-dependent enterocyte death (*n* = 6). **(A)** Representative intestinal TUNEL staining sections from the control group and the oxygen groups (FiO_2_ of 40, 60, and 80%) were exposed for 168 h (7 days). **(B)** The graph of the number of apoptotic cells in the control and the oxygen groups. **(C)** Western blotting and the graphs of Caspase-1 and GSDMD levels in the control and the oxygen groups. TUNEL, TdT-mediated dUTP-biotin nick end labeling; GSDMD, gasdermin-D. *P < 0.05; **P < 0.01; ***P < 0.001; ****P < 0.0001 (*P*-values: 80% of oxygen group vs. the control group).

Figure 2A shows the representative sections of TUNEL staining from the control and oxygen groups exposed for 168 h (7 days). We compared the number of apoptotic cells (Figure 2B) and expression of pyroptosis protein caspase-1 and GSDMD (Figure 2C) in the control group vs. the oxygen groups (*n* = 6 per exposure per time point) and showed the ANOVA analysis in Supplementary Table 2. The number of intestinal apoptotic cells increased after exposure to 80% of oxygen for 72 and 168 h, respectively (*P* = 0.009 and *P* < 0.0001; Figure 2B). Expression of GSDMD was elevated after exposure to 80% of oxygen for 24, 72, and 168 h, respectively (*P* = 0.011, *P* = 0.011, and *P* = 0.003), and the expression of caspase-1 was upregulated after exposure to 80% of oxygen for 24, 72, and 168 h, respectively (*P* = 0.006, *P* = 0.0004, and *P* < 0.0001; Figure 2C). Additionally, 40% of oxygen provoked intestinal cell apoptosis after exposure for 168 h (*P* = 0.012), and 60% of oxygen induced enterocyte apoptosis after exposure for 72 and 168 h, respectively (*P* = 0.015 and *P* < 0.0001).

### Hyperoxia Induces Time- and Dose-Dependent Gut Barrier Dysfunction and Endotoxemia

**Figure 3:**
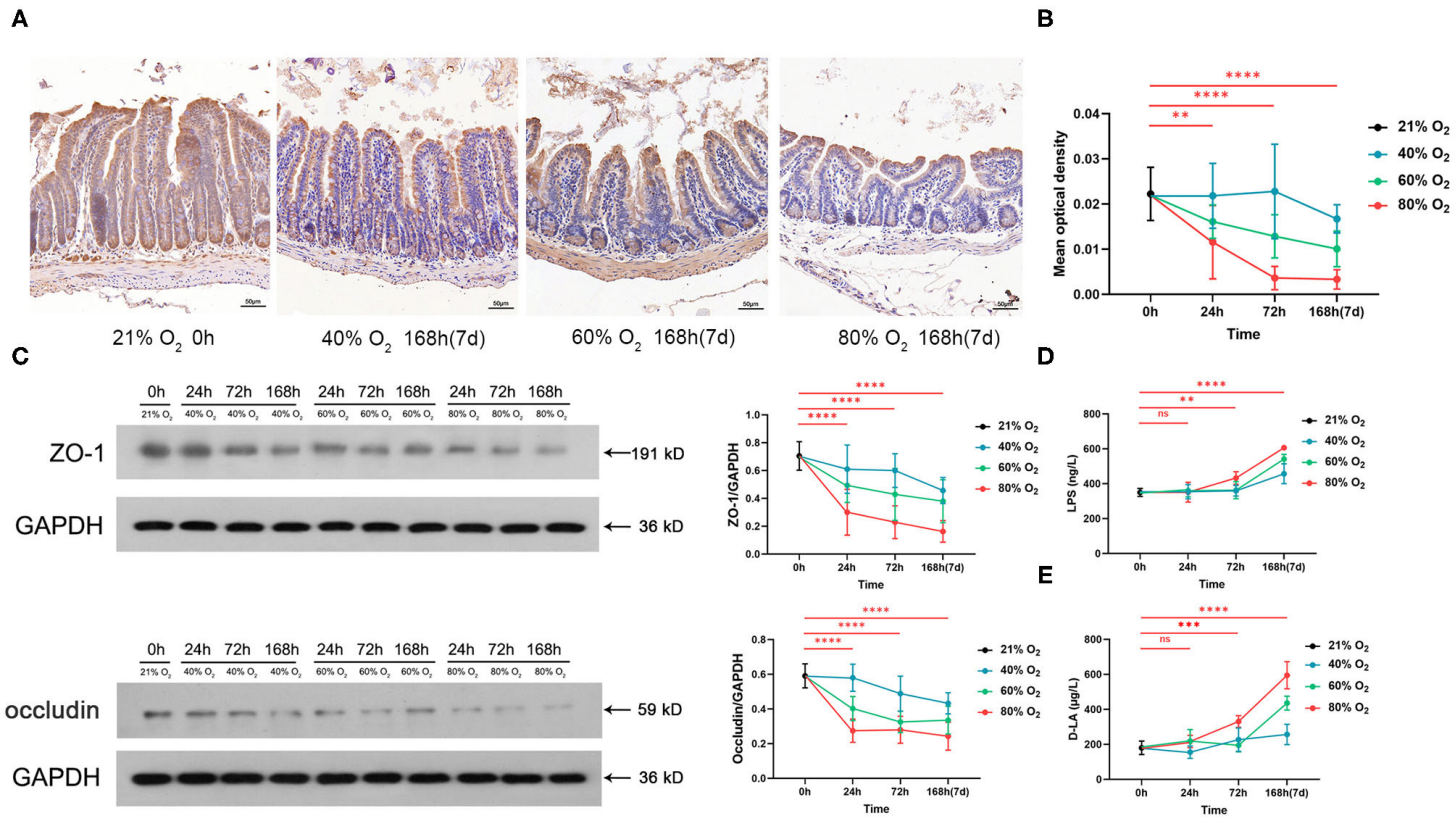
Hyperoxia induces time- and dose-dependent gut barrier dysfunction and endotoxemia (*n* = 6). **(A)** Representative intestinal immunohistochemical staining of ZO-1 sections from the control group and the oxygen groups (FiO_2_ of 40, 60, and 80%) exposed for 168 h (7 days). **(B)** The graph of the mean optical density of ZO-1 in the control and the oxygen groups. **(C)** Western blot and the graphs of ZO-1 and occludin levels in the control and the oxygen groups. **(D)** The graph of serum lipopolysaccharide (LPS) in the control and the oxygen groups. **(E)** The graph of serum D-LA in the control and the oxygen groups. LPS, lipopolysaccharide. **P < 0.01; ***P < 0.001; ****P < 0.0001 (*P*-values: 80% of oxygen group vs. the control group).

Figure 3A shows the representative sections of immunohistochemical staining of ZO-1 from the control group and the oxygen groups exposed for 168 h (7 days). We compared the expression of ZO-1 and occludin and the serum level of LPS in the control group vs. the oxygen groups (*n* = 6 per exposure per time point, Figures 3B–D), and the ANOVA analysis is shown in Supplementary Table 3. The mean optical density of ZO-1 decreased after exposure to 80% of oxygen for 24, 72, and 168 h, respectively (*P* = 0.00626, *P* < 0.0001, and *P* < 0.0001; Figure 3B). Expression of ZO-1 was downregulated after exposure to 80% of oxygen for 24, 72, and 168 h, respectively (all *P* < 0.0001), and expression of occludin was suppressed after exposure to 80% of oxygen for 24, 72, and 168 h, respectively (all *P* < 0.0001; Figure 3C). The serum level of LPS surged after exposure to 80% of oxygen for 72 and 168 h, respectively (*P* = 0.002 and *P* < 0.0001; Figure 3D). The serum level of D-LA increased after exposure to 80% of oxygen for 72 and 168 h, respectively (*P* = 0.0001 and *P* < 0.0001; Figure 3E). Overall, 40% of oxygen induced gut barrier dysfunction and endotoxemia after exposure for 168 h, and 60% of oxygen induced gut barrier dysfunction after exposure for 72 and 168 h, respectively, and provoked endotoxemia after exposure for 168 h.

### Hyperoxia Induces Time- and Dose-Dependent Gut Inflammatory Response and Oxidative Stress

**Figure 4:**
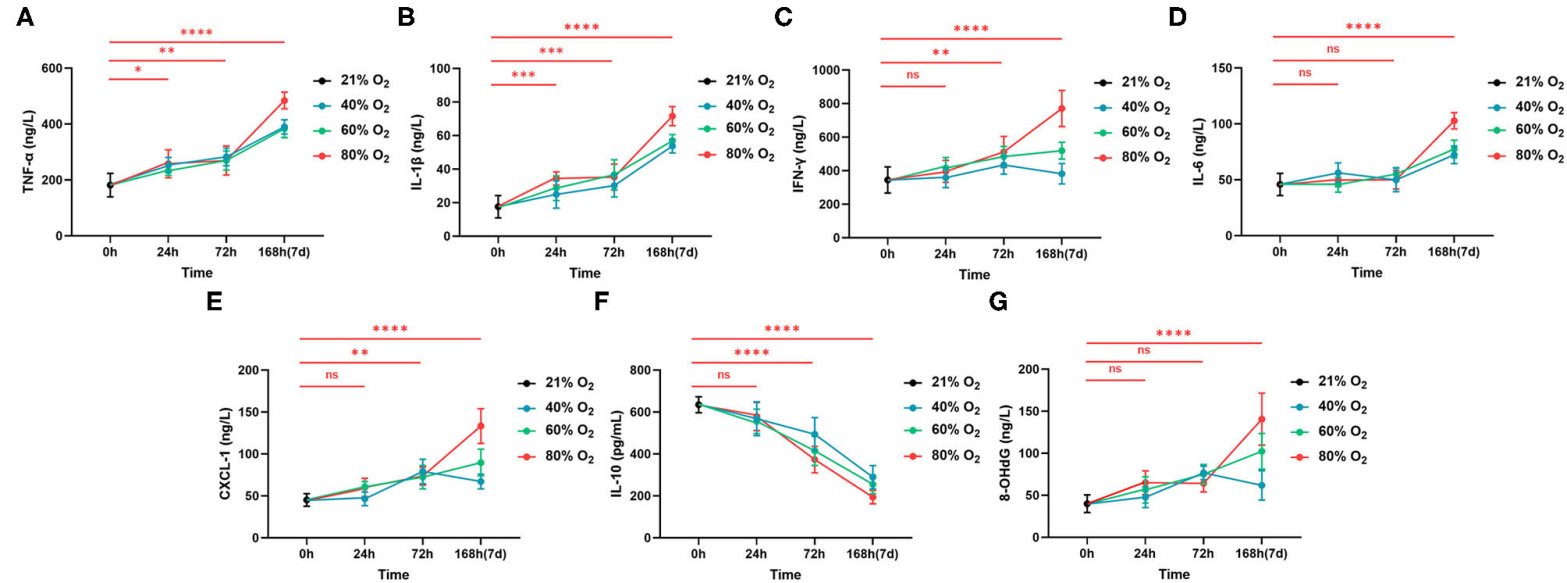
Hyperoxia induces time- and dose-dependent intestinal inflammation (*n* = 6). **(A)** The graph of concentrations of TNF-α. **(B)** The graph of concentrations of IL-1β. **(C)** The graph of concentrations of IFN-γ. **(D)** The graph of concentrations of IL-6. **(E)** The graph of concentrations of CXCL-1. **(F)** The graph of concentrations of IL-10. **(G)** The graph of concentrations of 8-OHdG. *P < 0.05; **P < 0.01; ***P < 0.001 ****P < 0.0001 (*P*-values: 80% of oxygen group vs. the control group).

Intestinal inflammatory cytokines and chemokines from the control group and oxygen groups (*n* = 6 per exposure per time point) are shown in Figure 4 and ANOVA analysis in Supplementary Table 4. Eighty percent of oxygen potentiates intestinal inflammation with increased TNF-α (24 h: *P* = 0.019, 72 h: *P* = 0.007, 168 h: *P* < 0.0001), IL-1β (24 h: *P* = 0.0004, 72 h: *P* = 0.0002, and 168 h: *P* < 0.0001), IFN-γ (72 h: *P* = 0.009, 168 h: *P* < 0.0001), IL-6 (168 h: *P* < 0.0001), and CXCL-1 (72 h: *P* = 0.004, 168 h: *P* < 0.0001; Figures 4A–E). However, 80% of oxygen decreased the level of IL-10 (72 and 168 h: all *P* < 0.0001; Figure 4F). Additionally, 80% of oxygen potentiated intestinal oxidative stress (8-OHdG, 72 h: *P* = 0.0002, 168 h: *P* < 0.0001; Figure 4G). Moreover, 40 and 60% of oxygen also induced more robust intestinal inflammation characterized by increased TNF-α, IL-1β and CXCL-1 and decreased IL-10, after exposure for 72 and 168 h, respectively.

### Gut Dysbiosis Induced by Hyperoxia

**Figure 5:**
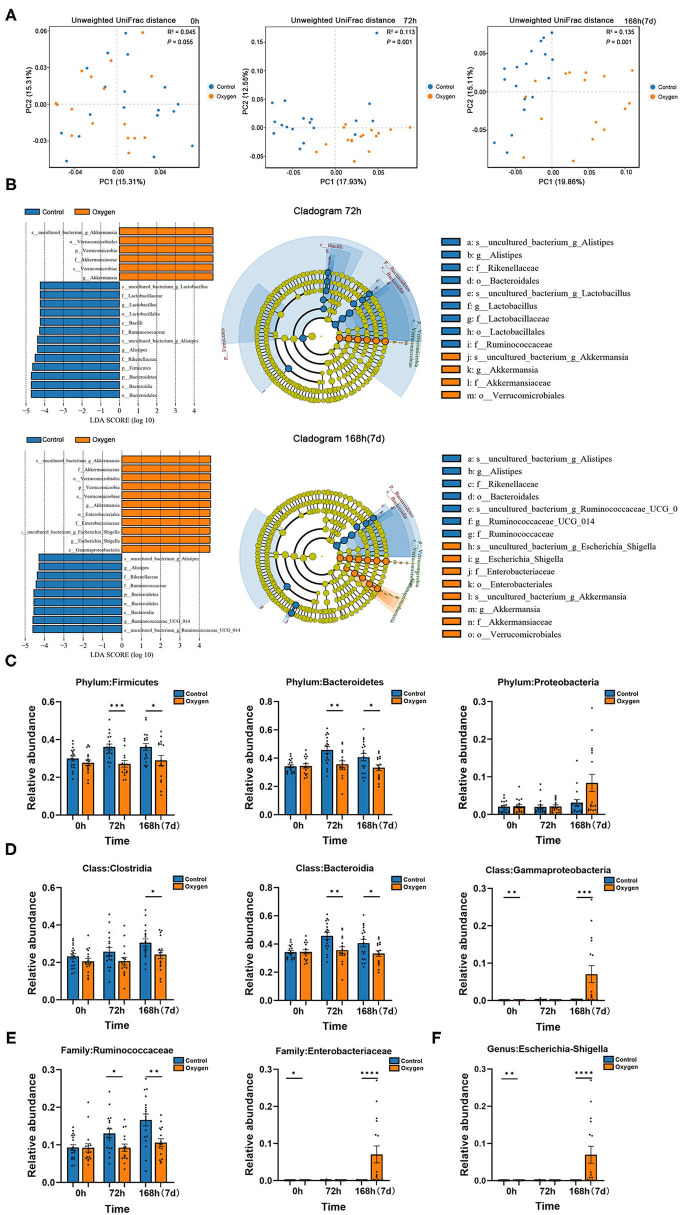
Hyperoxia alters gut microbiota in mice. **(A)** PCoA plots of the control groups (*n* =18) vs. the oxygen groups (*n* = 16) at 0, 72, and 168 h (7 days) based on Unweighted UniFrac distances. **(B)** LDA along with effect size measurements was applied to the enriched bacteria from the genus level to the phylum level in the control and oxygen groups at 72 h and 7 days, respectively. **(C–F)** The detailed comparison of the biomarkers of taxonomy in the control and oxygen groups at different time points (0, 72 h, and 7 days) at the Phylum, Class, Family, and Genus level, respectively. PCoA, principal co-ordinates analysis; LDA, linear discriminant analysis. *P < 0.05; **P < 0.01; ***P < 0.001; ****P < 0.0001.

The PCoA plots are presented in Figure 5A for the beta diversity analysis between the control groups and oxygen groups at time points of 0, 72, and 168 h (7 days), respectively. There was no significant difference (*R*^2^ = 0.045, *P* = 0.055) between the control group and oxygen group at baseline (0 h). However, significant differences were detectable at the durations of hyperoxia (72 h: *R*^2^ = 0.113, *P* = 0.001; 7 d: *R*^2^ = 0.135, *P* = 0.001). Further LDA analysis showed the enriched bacteria in the two groups at 72 h and 7 days, respectively (Figure 5B). At the early stage (72 h), hyperoxia diminished the obligate anaerobe in the oxygen group vs. the control group, such as Genus *Ruminococcaceae* (Class *Clostridia*, Phylum *Firmicutes*) and Class *Bacteroidia* (Phylum *Bacteroidetes*). However, at the late stage (7 days), hyperoxia not only diminished the obligate anaerobe but also enriched the facultative anaerobe (e.g., Genus *Escherichia-Shigella*: Family *Enterobacteriaceae*, Class *Gammaproteobacteria*, Phylum *Proteobacteria*) in the oxygen group. The detailed comparison of the biomarkers of taxonomy in the control and oxygen groups at different time points (0, 72, and 7 days) are shown in Figure 5C (Phylum), Figure 5D (Class), Figure 5E (Family), and Figure 5F (Genus).

### Transcriptome Analysis of Oxygen-Induced Gut Injury

**Figure 6:**
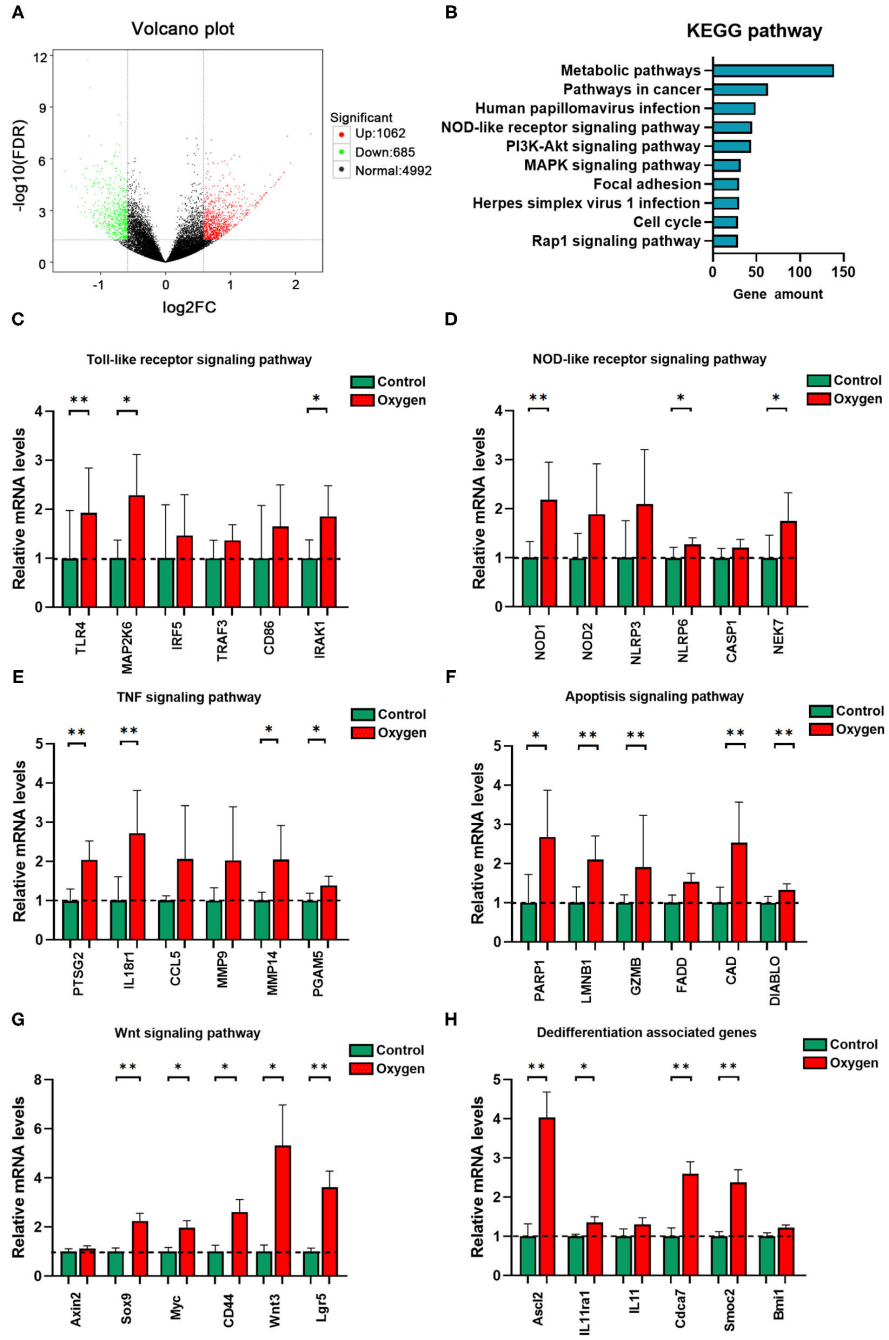
Transcriptome analysis of oxygen-induced gut injury (*n* = 6). **(A)** Volcano plot identifying DEGs between the oxygen and the control groups (Log_2_ FC ≥ 1.5, FDR < 0.05). **(B)** KEGG pathway analysis shows the top 10 significant pathways (*P* < 0.05) with the greatest amount of DEGs. **(C)** Relative mRNA levels of genes involved in the TLR signaling pathway. **(D)** Relative mRNA levels of genes involved in the NOD-like receptor signaling pathway. **(E)** Relative mRNA levels of genes involved in the TNF signaling pathway. **(F)** Relative mRNA levels of genes involved in the apoptosis signaling pathway. **(G)** Relative mRNA levels of genes involved in the Wnt signaling pathway. **(H)** Relative mRNA levels of genes involved in dedifferentiation. DEG, differentially expressed gene; FC, fold change; FDR, false discovery rate; KEGG, Kyoto Encyclopedia of Genes and Genomes; mRNA, messenger RNA; TLR, toll-like receptor; NOD, nucleotide-binding oligomerization domain. *P < 0.05; **P < 0.01.

There was a total of 1,747 DEGs identified in the oxygen group (FiO_2_ 80% for 14 days) in comparison to the controls while meeting the threshold of false discovery rate (FDR) <0.05 and log_2_ fold-change (FC) ≥ 1.5. Of these, 1,062 were upregulated DEGs, and 685 were downregulated (Figure 6A). The DEGs were annotated into the KEGG pathways, and the KEGG enrichment analysis produced 171 significant KEGG pathways (*P* < 0.05, Supplementary Table 5). The top 10 KEGG pathways with the most DEGs are presented in Figure 6B. Given the importance of innate immunity, inflammatory response, and cell death in oxygen-induced gut injury, we showed the DEGs involved in TLR (Figure 6C), NOD-like receptor (Figure 6D), TNF (Figure 6E), and apoptosis (Figure 6F) signaling pathways. In addition, we showed the Wnt signaling pathway (Figure 6G) and dedifferentiation genes (Figure 6H) involved in gut regenerative potential. The KEGG enrichment analysis revealed that the antioxidant signaling pathway (KEAP1-NRF2 signaling pathway) did not show any statistical significance (*P* > 0.05). However, some antioxidant genes of the gut tissues were downregulated under hyperoxia (Table 1).

**Table 1:** Differential expression analysis of gut antioxidant genes induced by 80% of oxygen.

**Gene symbol**	**Control**	**Oxygen**	**log_2_ FC**	***P*** -value^*^
	**(14 d, *n* = 6)**	**(14 d, *n* = 6)**		
Gpx4	247.70 ± 27.55	207.10 ± 35.95	−0.5369	0.0117^*^
Gpx7	2.45 ± 0.64	2.08 ± 0.53	−0.5261	0.0466^*^
Gstt1	15.33 ± 2.38	14.50 ± 1.68	−0.4052	0.0140^*^
Gstm5	12.33 ± 2.43	11.56 ± 1.98	−0.4026	0.0256^*^
Gsto1	257.00 ± 21.95	243.10 ± 26.22	−0.3853	0.0224^*^
Sod1	290.90 ± 10.84	283.20 ± 23.95	−0.3510	0.0236^*^

FPKM Values are expressed as mean ± SD. Differential expression analysis used with statistical significance set at ^*^P < 0.05. FPKM, Fragments Per Kilobase of exon model per Million mapped fragments; FC, Fold Change.

### Validation of Protein Levels by Western Blotting

**Figure 7:**
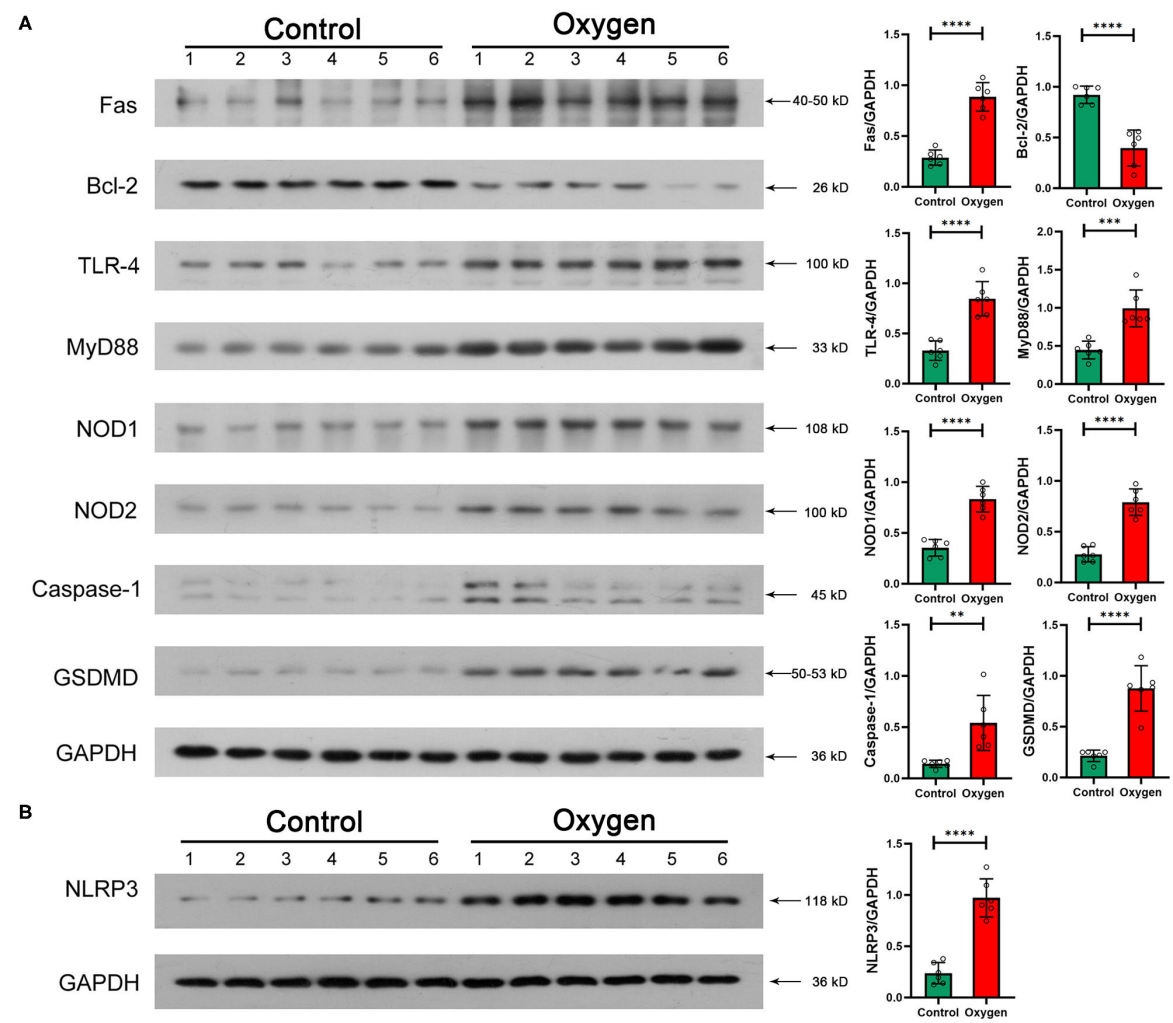
Essential protein levels of innate immune response and enterocyte death induced by hyperoxia (*n* = 6). **(A)** TLR-4, Myd88, NOD1/2, and NLRP3 protein levels in the gut tissue. Oxygen inhalation significantly increased the expression of TLR-4 (*P* < 0.0001), Myd88 (*P* = 0.0005), NOD1 (*P* < 0.0001), NOD2 (*P* < 0.0001), and NLRP3 (*P* < 0.0001). **(B)** Fas, Bcl-2, Caspase-1, and GSDMD protein levels in the gut tissue. Administered oxygen significantly increased the expression of Fas (*P* < 0.0001), Caspase-1 (*P* = 0.0048), and GSDMD (*P* < 0.0001) and decreased the expression of Bcl-2 (*P* < 0.0001). TLR, toll-like receptor; NOD, nucleotide-binding oligomerization domain; GSDMD, gasdermin-D. **P < 0.01; ***P < 0.001; ****P < 0.0001.

Western blotting was performed to validate essential intestinal protein levels in signaling pathways involved in innate immunity and cell death. Hyperoxia (FiO_2_ 80% for 14 d) promoted the expression of TLR-4 (*P* < 0.0001) and Myd88 (*P* = 0.0005) involved in TLR-4 signaling pathway as well as the expression of NOD1 (*P* < 0.0001), NOD2 (*P* < 0.0001) and NLRP3 (*P* < 0.0001) involved in the NOD-like receptor signaling pathway (Figure 7A). Additionally, hyperoxia led to apoptosis by increasing the level of Fas (*P* < 0.0001) and decreasing the level of Bcl-2 (*P* < 0.0001), and resulted in pyroptosis by elevating the level of caspase-1 (*P* = 0.0048) and GSDMD (*P* < 0.0001; Figure 7B).

## Discussion

It has been more than 200 years since the medical application of oxygen therapy in the late eighteenth century (35). Inhaled oxygen has been the most prescribed drug among millions of patients who are critically ill (36). However, supplementary oxygen usually exposes patients to arterial hyperoxia, leading to multiple organ injuries and poor clinical outcomes (37). The lungs are the first organs exposed to oxygen and are currently considered to be the most important targeted organ susceptible to hyperoxia (38). Yet, there is increasing evidence showing that hyperoxia systemically influences almost all human organs by increasing the dissolved oxygen in plasma and leading to oxidative stress (39, 40). The gut associated with the gut microbiome is the driver of systemic inflammation and MODS and is adversely influenced by critical illnesses (41). Thus, it is of clinical importance to explore the impact of hyperoxia on gut and gut microbiota.

Our study shows that time- and dose-dependent hyperoxia induces gut injury characterized by shortening of villus height, crypt depth, and a notable increase in enterocyte death. The IECs cover the intestine as a single layer and contribute to nutrient absorption, barrier function, and host immune defense against hazardous bacteria in the gut lumen (42). The natural turnover of the gut epithelium is as short as 4–5 days, and the death of IECs is tightly controlled (43). IEC apoptosis induced by hyperoxia (Figure 1B) underlies the gut epithelial atrophy, indicating the dysfunction of gut absorption, barrier, and immunity. In addition, there are less paneth cells in the hyperoxia groups vs. the control group. Paneth cells play a key role in the homeostasis of the small intestinal epithelium, and the loss or destruction of these cells may lead to adverse consequences including dysfunction of clearance of bacterial pathogens and disturbances of stem cells (44, 45). Our study also shows that hyperoxia can lead to an elevated level of intestinal lysozyme. The lysozyme-mediated digestion of PGN leads to the activation of multiple innate immune receptor families that contribute to proinflammatory responses (NOD, TLR, and inflammasomes) (46). Pyroptosis is an important form of inflammatory cell death that is initiated by an assembly of inflammasomes, especially NLRP3 from the NOD-like receptor family (47, 48). Caspase-1 is activated by the NLRP3 inflammasome and induces proinflammatory cytokines (IL-1β and IL-18) and the pore-forming protein GSDMD, finally triggering pyroptosis (45). Our study shows that pyroptosis is another type of enterocyte death induced by hyperoxia.

Adjacent IECs form tight junctions (TJs) to provide a physical intestinal barrier that regulates the paracellular movement of vital substances such as water, ions, and various small molecules and limits the permeation of luminal pathogens and toxins, such as LPS (49). ZO-1 and occludin play a critical role in the assembly and function of TJs (50). Furthermore, ZO-1 is a biomarker of gut barrier dysfunction in septic patients (51). D-LA can also be used as a biomarker for intestinal permeability (52) and was elevated by hyperoxia. Increased gut permeability leads to LPS endotoxemia, which is associated with prolonged ICU hospital stays, a high frequency of organ dysfunction, and systemic inflammation (53). Additionally, LPS recognition by TLR-4 leads to the release of proinflammatory cytokines and cell death. This effect influences cardiac function, capillary permeability, vascular resistance, and disrupts cellular metabolism. Our study confirms that hyperoxia rapidly induces gut-derived endotoxemia, which may contribute to oxygen-induced injuries in remote organs (e.g., the lung) by the TLR pathway. It is indicated that hyperoxia may exert an influence on the pathophysiology and outcomes of critical illnesses through oxygen-induced gut barrier dysfunction and endotoxemia.

We also demonstrated intestinal inflammation induced by hyperoxia with histopathological examinations (Figure 1C) and quantitative analysis of cytokines. Hyperoxia elevated the levels of proinflammatory cytokines including TNF-α, IFN-γ, and IL-1β. We suggest that the enhanced proinflammatory response may suppress the intestinal host defense and modulate the acute gut injury induced by hyperoxia (54). Additionally, hyperoxia decreases the release of IL-10, which is considered to be the most potent anti-inflammatory cytokine to date. Decreased IL-10 is also reported in oxygen-induced lung injury in which supplemental IL-10 demonstrates a therapeutic effect (55). IL-6 has both anti- and proinflammatory properties (56) and is upregulated by hyperoxia. Our study shows that CXCL-1 is induced by hyperoxia and indicates that gut neutrophilia (Figure 1C) is an important pathophysiological process in hyperoxia-induced gut injury. In addition, these findings show that TNF-α and IL-1β are the first proinflammatory markers to rise, indicating the important role in promoting the gut inflammation cascade induced by hyperoxia. In addition, the infiltrates of macrophages induced by oxygen may contribute to the increased TNF-α and IFN-1β in the gut mucosa. Also, hyperoxia elevates the level of 8-OHdG and decreases the expression of antioxidant genes in the gut tissues. These results suggest that the enhanced oxidative stress induced by administered oxygen leads to impaired antioxidant potential and oxidative injury in the gut.

Our study shows that high concentrations of oxygen (80%) provoke gut injury and endotoxemia after short exposure (24–72 h), and low and moderate concentrations of oxygen (40 and 60%) also induce gut injury and endotoxemia after prolonged exposure (72–168 h). These findings challenge the previous general agreement that high concentrations of oxygen (>70%) result in harmful effects after prolonged administration (57). Currently, low concentration oxygen (e.g., FiO2 of 40%) is routinely administered to patients in ICUs (58). However, this study suggests that even low concentrations of oxygen are also deleterious to the gut (Table 2). We found that both oxygen concentration and duration of exposure contributed to the enhanced gut injury on the basis of ANOVA. Overall, it is indicated that hyperoxia can rapidly induce gut injury and endotoxemia.

**Table 2:** ANOVA analysis and Dunnett's *post hoc* test of time-dependent gut histopathological injury, enterocyte death, gut barrier dysfunction, endotoxemia, and gut inflammation induced by 40% of oxygen.

	**0 h (*n* = 6)**	**24 h (*n* = 6)**	**72 h (*n* = 6)**	**168 h (7 d) (*n* = 6)**	***P*** -value^*^		
					***P*** **(0 vs. 24 h)**	***P*** **(0 vs. 72 h)**	***P*** **(0 vs. 7 d)**
Crypt depth (μm)	74.91 ± 8.48	70.56 ± 5.85	63.10 ± 10.11	56.58 ± 6.71	0.6681	0.0470^*^	0.0020^*^
TUNEL positive/100 cells	0.59 ± 0.50	0.72 ± 0.37	0.99 ± 0.24	1.47 ± 0.70	0.9245	0.3381	0.0124^*^
ZO-1/GAPDH	0.71 ± 0.17	0.61 ± 0.17	0.60 ± 0.12	0.46 ± 0.09	0.4383	0.3601	0.0077^*^
occludin/GAPDH	0.59 ± 0.07	0.58 ± 0.08	0.49 ± 0.10	0.43 ± 0.06	0.9901	0.0869	0.0062^*^
LPS (ng/L)	350.43 ± 22.68	354.46 ± 40.66	359.57 ± 29.94	457.15 ± 56.92	0.9961	0.9586	0.0004^*^
TNF-α (ng/L)	181.84 ± 42.07	253.55 ± 27.06	282.50 ± 31.37	390.39 ± 25.57	0.0027^*^	<0.0001^*^	<0.0001^*^
IL-1β (ng/L)	17.64 ± 6.69	24.93 ± 8.12	30.22 ± 6.70	53.77 ± 4.10	0.1662	0.0095^*^	<0.0001^*^
IL-6 (ng/L)	45.90 ± 9.92	56.36 ± 8.78	49.92 ± 10.52	72.48 ± 7.86	0.1584	0.7968	0.0002^*^
CXCL-1 (ng/L)	45.28 ± 7.47	46.65 ± 8.28	79.21 ± 14.59	67.21 ± 8.77	0.9910	<0.0001^*^	0.0036^*^
IL-10 (pg/mL)	635.14 ± 38.13	568.12 ± 80.59	494.51 ± 79.03	289.65 ± 55.30	0.2153	0.0038^*^	<0.0001^*^

Values are expressed as mean ± SD. One-Way ANOVA used with statistical significance set at ^*^P < 0.05.

Molecular oxygen in the environment dramatically influences the physiology and ecology of bacteria (12). The gut harbors a huge and complex community of species of bacteria, most of which are obligate anaerobe, especially the classes *Clostridia* (Phylum *Firmicutes*) and *Bacteroidia* (Phylum *Bacteroidetes*) (59). The partial pressure of oxygen is extremely low in the intestinal lumen (<1 mmHg) and is increased after oxygen inhalation (12). Recent animal studies reveal that hyperoxia has the potential to modify the structure of gut microbiota (14–16). However, these findings are limited by the high heterogeneity, as a result of different sample sizes, animal species, animal ages, exposure durations, and hyperoxia modeling methods (13–17). The strength of our study is that we explore the oxygen-induced gut dysbiosis with a relatively large sample size (34 mice per time point) at different stages of exposure to hyperoxia (0, 72 h, and 7 days, respectively).

We find out that administered oxygen dynamically changes the composition of the gut microbiome. At the early stage (72 h), hyperoxia depletes the oxygen-intolerant bacteria, such as Genus *Ruminococcaceae* (Class *Clostridia*) and Class *Bacteroidia*. However, there is a great expansion of oxygen-tolerant bacteria (*Enterobacteriaceae*: Class *Gammaproteobacteria*, Phylum *Proteobacteria*) associated with the depletion of oxygen-intolerant bacteria, at the late stage (7 days). *Enterobacteriaceae* is a gram-negative facultative anaerobe that is conventionally colonized in the gut lumen and has the potential to be pathogenic (60). It is the hallmark of gut dysbiosis and the common source of both community and hospital-acquired infections in human beings (55). In addition, the abundance of *Enterobacteriaceae* predicts a high mortality rate in patients who are critically ill (61). These findings indicate that the oxygen-induced expansion of *Enterobacteriaceae* in the gastrointestinal tract may play critical roles in the progression of critical illnesses and MODS. Last, the 16S rRNA sequencing analysis reveals that Genus *Escherichia -Shigella* (Family *Enterobacteriaceae*) is the biomarker bacteria at the genus level. *Escherichia coli* remains one of the most common pathogenic bacteria in clinical settings (62). We infer that *Escherichia coli* may constitute the most abundant species in the oxygen-induced expansion of *Enterobacteriaceae*. Further metagenomic analysis and isolation and cultivation of bacteria are needed to identify the specific species in oxygen-induced gut dysbiosis, in the future.

**Figure 8:**
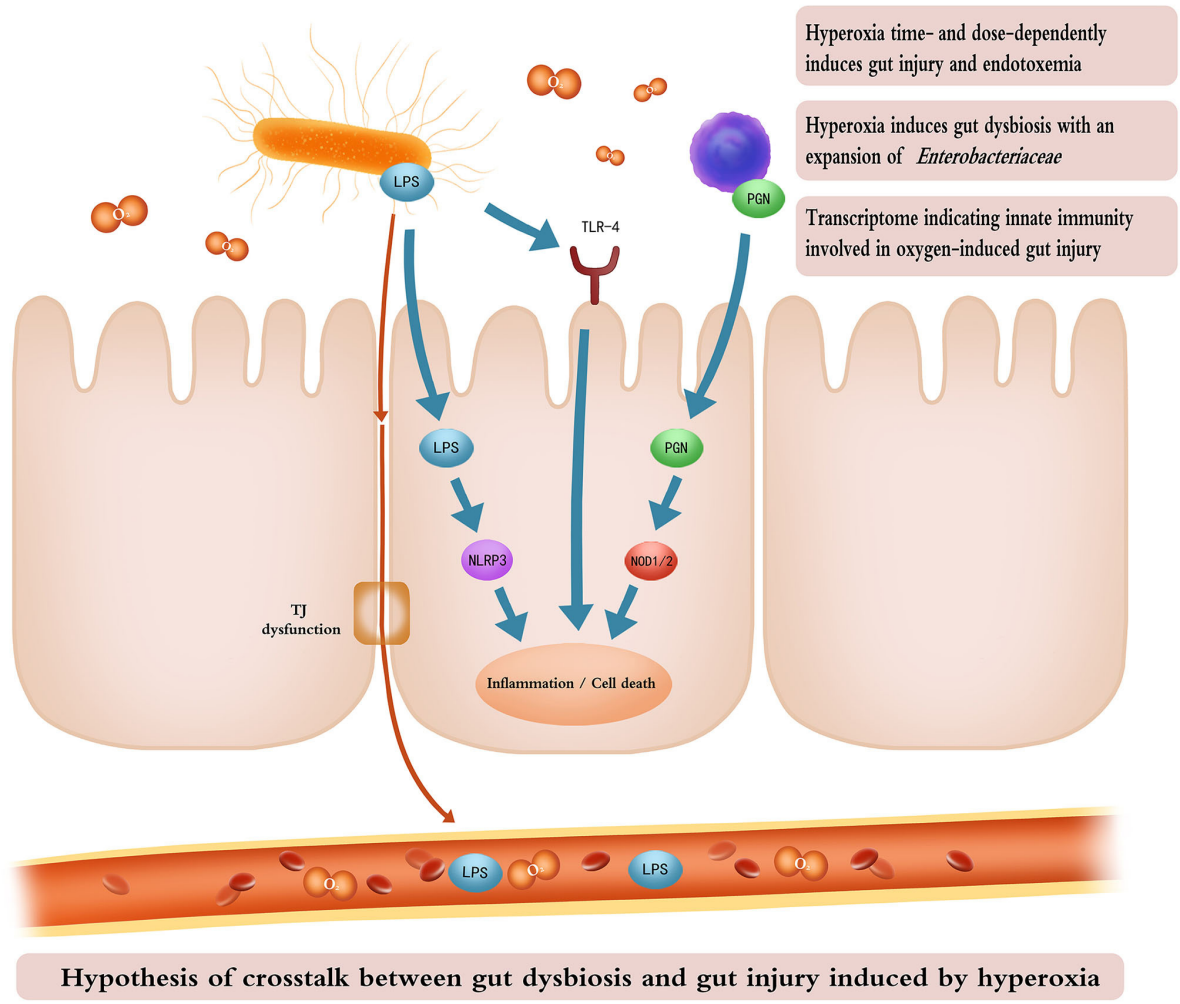
Cartoon illustrating the hypothesis of crosstalk between gut dysbiosis and gut injury induced by hyperoxia. Time- and dose-dependent hyperoxia induces intestinal inflammation and enterocyte death and TJ dysfunction. Inhaled oxygen promotes the expansion of *Enterobacteriaceae* which releases LPS through impaired TJ into the bloodstream, resulting in endotoxemia. LPS from gram-negative bacteria and PGN from gram-positive bacteria activate innate immunity (TLR-4, NLRP3, and NOD signaling pathways), leading to gut inflammation and cell death. The cartoon was painted by Yuxin Wang for this study. TJ, tight junction; LPS, lipopolysaccharide; PGN, peptidoglycan; TLR-4, toll-like receptor 4; NLRP3, recombinant NLR Family, pyrin domain containing protein 3; NOD, nucleotide-binding oligomerization domain.

A recent study suggests that the lung and gut microbiota contribute to oxygen-induced lung injury (16). Hyperoxia provokes both gut dysbiosis and gut injury. We hypothesize that the gut microbiota may also play a fundamental role in the development of oxygen-induced gut injury. The transcriptome associated with immunoblotting analysis revealed that TLR and NOD-like receptor signaling pathways are involved in the hyperoxia-induced gut injury. LPS and PGN are the major components of the cell wall of gram-negative (e.g., *Enterobacteriaceae*) and gram-positive bacteria, respectively (63, 64). They both play a key role in host–bacteria interactions through innate immunity and contribute to bacterial sepsis in patients who are critically ill (65). LPS recognition by the transmembrane TLR-4 and the cytoplasmic NLPR3 initiates an immune response, resulting in inflammation and cell apoptosis, and pyroptosis (66, 67). NOD1 and NOD2 sense intracellular bacterial PGN and lead to the activation of innate immunity and proinflammatory response (68). Consequently, we hypothesize that gut microbes may modulate the oxygen-induced gut injury through an innate immune response, especially TLR-4 and NOD-like receptor signaling pathways (Figure 8). Wnt signaling pathway and the dedifferentiation activity contribute to intestinal regeneration (69, 70), and are activated by oxygen-inhalation. Therefore, hyperoxia induces gut injury but enhances the potential of intestinal regeneration.

**Figure 9:**
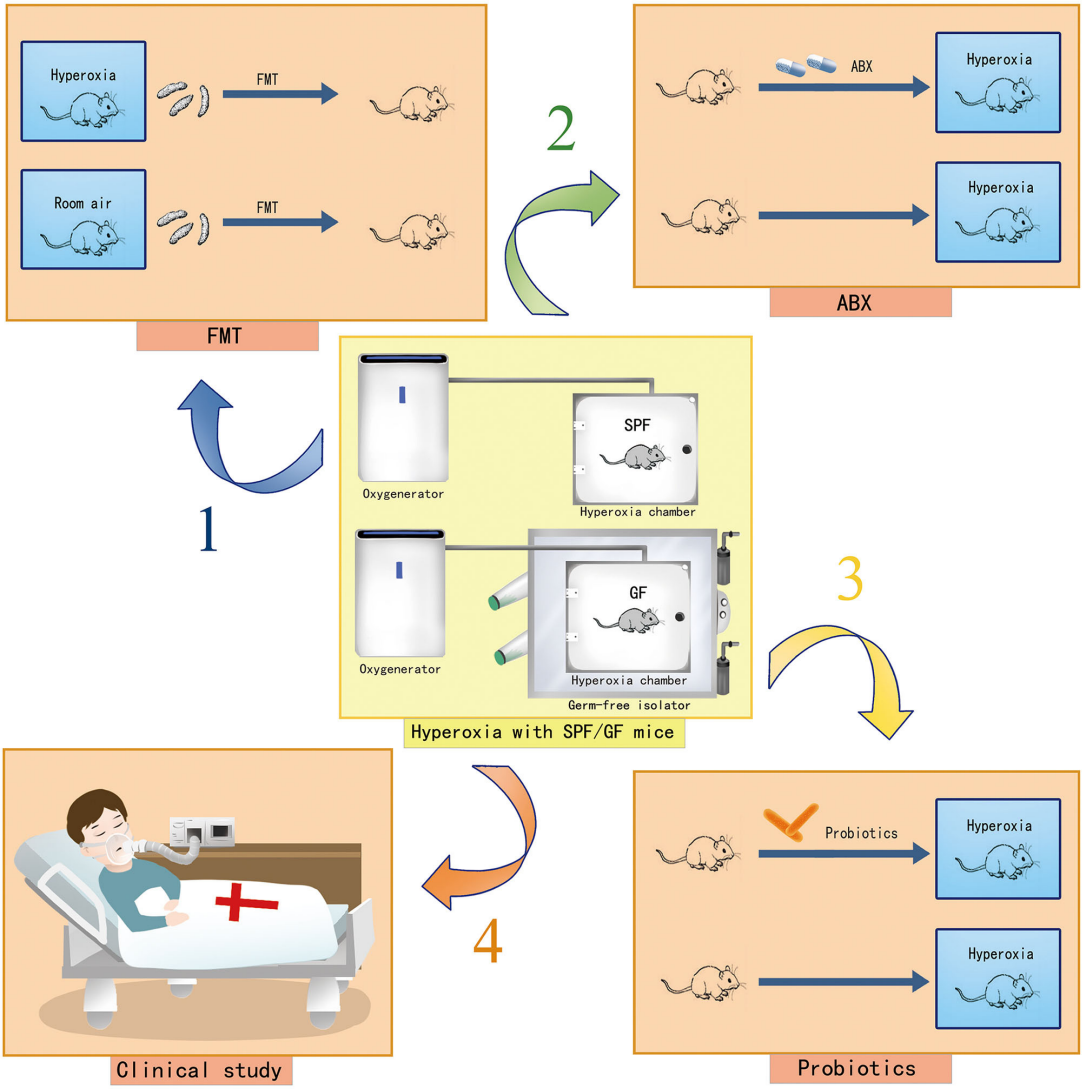
Cartoons showing the study protocol to explore the role of gut dysbiosis in hyperoxia-induced gut injury. Hyperoxia modeling with GF mice in a GF isolator and hyperoxia modeling with SPF mice are presented. Arrow 1 indicates hyperoxia modeling with FMT to identify the targeted bacteria involved in an oxygen-induced gut injury. Arrow 2 indicates hyperoxia modeling with ABX mice to explore the impact of systemic antibiotic treatment on the severity of the oxygen-induced gut injury. Arrow 3 indicates hyperoxia modeling with probiotics to explore the protective effect of probiotics on the oxygen-induced gut injury. Arrow 4 indicates the translational research in clinical settings to assess the impact of conservative oxygen therapy vs. liberal oxygen therapy on gut microbiome and outcomes of patients in ICU. GF, germ-free; SPF, specific pathogen free; FMT, fecal microbiota transplantation; ABX, antibiotic treated; ICU, intensive care unit.

There are several limitations to this study. Hyperoxia may induce gut injury at a super early stage. Our experimental approach did not investigate the oxygen-induced gut injury after exposure of 6–12 h. We explored the impact of hyperoxia on the small intestine, which is the longest part of the digestive tract that plays a critical role in nutrient digestion and absorption. Yet, we did not clarify the oxygen-induced injury in the large intestine which contains >70% of the microorganisms found in the body and accounts for most gut diseases, such as colon cancer and inflammatory bowel disease (71). Additionally, the profound impact of hyperoxia on the gut has not been verified in clinical settings, given the species difference between humans and mice. Last, we did not determine the crosstalk between gut microbiota and gut injury under hyperoxia. Here, we present the further study protocol including hyperoxia modeling experiments with germ free (GF) mice, fecal microbiota transplantation (FMT), antibiotic-treated (ABX) mice, and probiotics-intervention as well as the translational study in clinical settings assessing the impact of oxygen therapy (liberal vs. conservative oxygen therapy) on the gut microbiome in patients who are critically ill (Figure 9).

Our study demonstrates that hyperoxia rapidly induces gut injury and gut dysbiosis with a marked expansion of *Enterobacteriaceae* and proposes innate immunity involved in oxygen-induced gut injury through transcriptome analysis. Further clinical and experimental studies are needed to identify the following: (1) the impact of hyperoxia-induced gut injury and gut dysbiosis on sepsis and MODS; and (2) the role of gut microbiota in hyperoxia-induced gut injury and its underlying mechanism. GF and FMT mouse models may shed light on the triangular relationship among hyperoxia, gut injury, and gut microbiota. A deeper understanding of host-microbiota interactions may promote better therapeutic strategies for the prevention and treatment of oxygen-induced organ impairment in the future.

## Conclusion

Inhaled oxygen induces marked gut injury associated with endotoxemia in a time- and dose-dependent manner. A short exposure (24–72 h) to high concentrations of oxygen (80%) rapidly leads to gut injury and endotoxemia. Low and moderate concentrations of oxygen (40 and 60%) also cause gut injury and endotoxemia after prolonged exposure (72–168 h). Hyperoxia provokes gut dysbiosis with a great expansion of *Enterobacteriaceae*. Transcriptome analysis reveals that innate immunity (TLR and NOD-like receptor signaling pathways) is involved in an oxygen-induced gut injury. Further studies are needed to understand the role of gut microbiota in oxygen-induced gut injury and to elucidate the effect of innate immunity in the crosstalk between gut and gut microbiota.

## Data Availability Statement

The original contributions presented in the study are publicly available. These data can be found here: https://ngdc.cncb.ac.cn/gsa/browse/CRA004276 and https://ngdc.cncb.ac.cn/gsa/browse/CRA004740.

## Ethics Statement

The animal study was reviewed and approved by Committee on Animal Care and Use of Ethical Committee of Zunyi Medical University.

## Author Contributions

YL and YT reviewed the literature, performed the study, and contributed to manuscript drafting. JX and YihH performed the statistical analysis and contributed to manuscript drafting. WZ interpreted the results of the experiments and drafted the manuscript. ZJ performed the biochemical analysis and drafted the manuscript. YL, HL, and YinH performed bioinformatics and statistical analysis for 16S rRNA. MC, WZ, and ZX reviewed the literature and were responsible for the important intellectual contents of the manuscript. All authors issued final approval for the version to be submitted.

## Funding

This study received financial support from the Guizhou Science and Technology Department ([2020]4Y199, [2020]1Z061, [2019]2824, [2021]YIBAN441), the National Natural Science Foundation of China (82160370), and the Department of Health of Guizhou Province (gzwjkj2020-1-021).

## Conflict of Interest

The authors declare that the research was conducted in the absence of any commercial or financial relationships that could be construed as a potential conflict of interest.

## Publisher's Note

All claims expressed in this article are solely those of the authors and do not necessarily represent those of their affiliated organizations, or those of the publisher, the editors and the reviewers. Any product that may be evaluated in this article, or claim that may be made by its manufacturer, is not guaranteed or endorsed by the publisher.

## Supplementary Material

Supplementary Figure 1 Increased level of intestinal lysozyme induced by hyperoxia (FiO_2_ 80% for 7 d).

Supplementary Table 1 ANOVA or non-parametric analysis of time- and dose-dependent gut histopathological injury induced by hyperoxia.

Supplementary Table 2 ANOVA analysis of time- and dose-dependent gut cell death induced by hyperoxia.

Supplementary Table 3 ANOVA analysis of time- and dose-dependent gut barrier dysfunction and endotoxemia by hyperoxia.

Supplementary Table 4 ANOVA analysis of time- and dose-dependent gut inflammation and oxidative stress induced by hyperoxia.

Supplementary Table 5 KEGG enrichment analysis of gut injury induced by hyperoxia.
