# Supplementary material for: Hyperoxia Provokes Time- and Dose-Dependent Gut Injury and Endotoxemia and Alters Gut Microbiome and Transcriptome in Mice
Source: Front Med (Lausanne). 2021 Nov 17;8:732039. doi: 10.3389/fmed.2021.732039 (PMC8635731; doi:10.3389/fmed.2021.732039)
Supplement: Supplementary Figure 1 — Increased level of intestinal lysozyme induced by hyperoxia (FiO2 80% for 7 d). [file Data_Sheet_1.PDF]

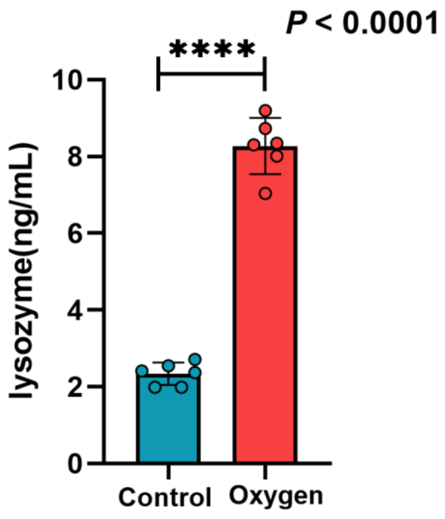

Supplementary Figure S1. Increased level of intestinal lysozyme induced by hyperoxia (FiO_2 80% for 7 d).
